# Cloning and expression of N22 region of Torque Teno virus (TTV) genome and use of peptide in developing immunoassay for TTV antibodies

**DOI:** 10.1186/1743-422X-11-96

**Published:** 2014-05-20

**Authors:** Dhananjay Singh Mankotia, Mohammad Irshad

**Affiliations:** 1 Clinical Biochemistry Division, Department of Laboratory Medicine, All India Institute of Medical Sciences, New Delhi 110029, India

**Keywords:** Torque teno virus, Cloning, Immunoassay

## Abstract

**Background:**

Torque Teno Virus (TTV) is a DNA virus with high rate of prevalence globally. Since its discovery in 1997, several studies have questioned the role of this virus in causing disease. However, it still remains an enigma. Although methods are available for detection of TTV infection, there is still a need for simple, rapid and reliable method for screening of this virus in human population. Present investigation describes the cloning and expression of N22 region of TTV-genome and the use of expressed peptide in development of immunoassay to detect anti-TTV antibodies in serum. Since TTV genotype-1 is more common in India, the serum positive for genotype-1 was used as source of N22 for expression purpose.

**Methods:**

Full length N22 region of ORF1 from TTV genotype-1 was amplified and cloned in pGEM®-T Easy vector. After cloning, the amplicon was transformed and expressed as a fusion protein containing hexa-histidine tag in pET-28a(+) vector using BL21 *E. coli* cells as host. Expression was conducted both in LB medium as well as ZYP-5052 auto-induction medium. The expressed peptide was purified using metal-chelate affinity chromatography and used as antigen in developing a blot immunoassay.

**Results:**

Analysis of translated product by SDS-PAGE and western blotting demonstrated the presence of 25 kDa polypeptide produced after expression. Solubility studies showed the polypeptide to be associated with insoluble fraction. The use of this peptide as antigen in blot assay produced prominent spot on membrane treated with sera from TTV-infected patients. Analysis of sera from 75 patients with liver and renal diseases demonstrated a successful implication of N22 polypeptide based immunoassay in screening sera for anti-TTV antibodies. Comparison of the immunoassay developed using expressed N22 peptide with established PCR method for TTV-DNA detection showed good coherence between TTV-DNA and presence of anti-TTV antibodies in the sera analysed.

**Conclusions:**

This concludes that TTV N22 region may be expressed and safely used as antigen for blot assay to detect anti-TTV antibodies in sera.

## Background

TTV is a small and non-enveloped virus with a circular, single-stranded and negative sense DNA genome of ~3.8 kb [1,2]. It was identified from the serum of a Japanese patient with post-transfusion non-A-E hepatitis by representational difference analysis [3]. TTV was found to infect both healthy and diseased individuals with some possible role in unexplained hepatitis. Initial studies revealed a higher frequency of TTV infection in patients with fulminant hepatitis, chronic liver disease of unknown aetiology and haemophiliac patients as compared to that in blood donors. However, later studies suggested that TTV is probably not the causative agent of acute sporadic hepatitis [4,5].

TTV genome was sequenced by Okamoto *et al.*[1] almost entirely on the prototype isolate TA278 encompassing 3739 nucleotides, and was temporarily labelled to be a linear DNA. However, subsequent studies [2] with the GH1 isolate and TA278 isolate [6] identified a GC-rich missing link of about 100 nucleotides in TTV genome. Thus, TTV is an unenveloped virus with a particle size of 30–50 nm, whose genome consists of a circular and single stranded DNA molecule of negative polarity of about 3.8 kb length [2]. The sequence of TTV genome is extremely heterogeneous. TTV isolates have been classified into five major phylogenetic groups with ~60% nucleotide sequence variability among them [7]. The sequence heterogeneity of the TTV genome, however, is more complex. One report described 16 TTV genotypes [8] whereas another study identified 5 additional TTV genetic groups [9]. Genogroup 3 contains the highest number of TTV isolates described till now with genogroup 2 and 4 to be less prevalent [10,11]. Full length sequences from several isolates of genotype 1–23 have been deposited in GenBank. Most genotypes hitherto sequenced contain 2–3 open reading frames (ORFs). ORF1 encodes putative capsid protein whereas ORF2 encodes non-structural proteins [12,13].

TTV infection is diagnosed on the basis of TTV-DNA detection by PCR. However, due to high sequence variation, it is difficult to find a universal primer set for all existing TTV-genotypes [9]. Detection of TTV by PCR method is also hindered due to presence of low viral load in sera, which requires the use of sensitive and reliable PCR protocols, that could differ among laboratories [5,12]. Immuno-precipitation experiments in the past have suggested the existence of TTV-specific antibodies. Prevalence of IgM class antibodies in sero-infected persons was revealed by use of an IgM-capture method combined with PCR [14]. Until now, only few reports have described the detection of anti-TTV antibodies and these studies have used either fragments of the ORF1-encoded protein or crude TTV particles as antigens [5,12,15]. However, these techniques are not useful for large-scale and/or routine screening and PCR detection, with all its limitations, is still used [5,12]. In spite of several means available to diagnose TTV infection, there is still a need of a simple and economic assay for its use in routine diagnostic laboratories for early diagnosis of this infection.

Present study describes the cloning, expression of TTV sub-genome (N22 region) and development of a simple immunoassay to detect presence of anti-TTV antibodies in human sera. The N22 region from ORF1 of TTV genome was cloned and recombinant protein was expressed in *E. coli.* The expressed translational product was used as antigen to develop blot assay for detection of antibodies directed against TTV. This immunoassay was used to find the prevalence of anti-TTV antibodies in a panel of sera from patients with wide variety of liver and renal diseases as well as healthy individuals.

## Results

**Figure 1:**
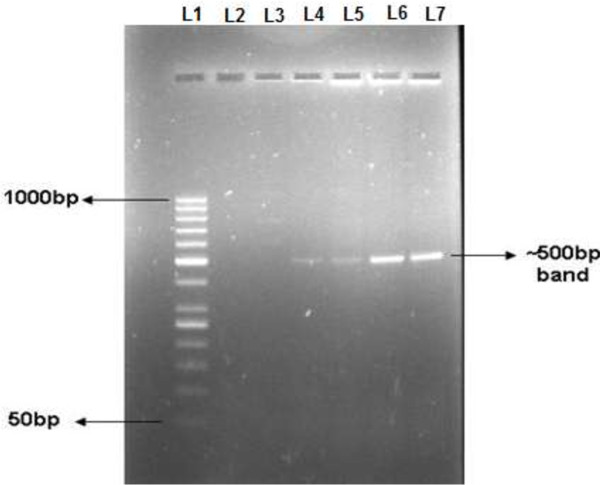
**Amplification of N22 region of TTV genotype-1.** N22 region was amplified using genotype specific primers. 1.2% agarose gel picture shows ~500 bp band corresponding to N22 region of genotype-1. Lane 1: 50 bp DNA marker; Lane 2 and 3: No template control; Lane 4–7: Bands of ~500 bp corresponding to N22 region amplified from TTV-DNA positive sera.

In the first phase, sera from patients with liver and renal diseases were analysed for detection of TTV-DNA using polymerase chain reaction (PCR). The sera found positive for TTV-DNA were subjected to TTV-genotyping by Restriction Fragment Length Polymorphism (RFLP) technique. Sera samples positive for TTV genotype-1 were used as the source of genotype-1 and chosen for amplification of N22 region and its subsequent expression studies. The preference of genotype-1 is based on its high prevalence in Indian populations [16]. N22 region of ORF1 from TTV genotype-1 was amplified using specific primers corresponding to nucleotide sequence 1847–2346 selected from known TTV genotype-1 isolate JA-20. The results of amplification by PCR demonstrated a 500 bp band on agarose gel (Figure 1).

The above amplicon of N22 region was cloned in pGEM^®^-T Easy vector and transformed in *E. coli* DH5α cells. Positive colonies were selected for confirmation of insertion by colony PCR. The insertion was also validated by sequence analysis where cloned product was sequenced bi-directionally and matched with known isolates of TTV genotype-1 N22 region using BLAST program of NCBI. The sequence matching of cloned N22 region revealed 97% identity with JA20 isolate of TTV. In addition to sequence analysis, the sequence of product was also analysed by constructing phylogenetic tree using NJ method. This also produced positive results.

After confirmation of cloning, the amplicon was generated from the clone and subsequently sub-cloned in pET-28a(+) vector allowing the expression of recombinant protein as fusion protein with a hexa-histidine tag. N22 region was re-amplified using primers containing restriction sites for *BamH*I and *Sac*I enzymes. The 500 bp amplicon obtained was digested with restriction enzymes *BamH*I and *Sac*I simultaneous with vector pET-28a(+) and then ligated using T4 DNA Ligase. The ligated product was transformed and positive colonies were selected for confirmation of insertion by colony PCR. The colony PCR produced a ~500 bp band on agarose gel confirming the insertion of amplicon in pET-28a(+) expression vector (Additional file 1: Figure S1). The extraction of recombinant plasmid from cultured homogeneous colony, followed by its restriction digestion and then run on gel demonstrated presence of a 500 bp and 5.7 kb bands on gel corresponding to released insert and linearised vector backbone, respectively. A complete battery of experiments including colony PCR, restriction digestion and sequence analysis were used to confirm the insertion in expression vector. In fact, confirmation of insertion of required target region has significance to avoid all future chances of error during expression step.

Expression was conducted in LB medium under variable conditions of incubation time, temperature and IPTG concentration. The results of SDS-PAGE demonstrated the presence of 25 kDa recombinant protein. The important observations made in this step were : a very low yield of peptide and very little effect of increasing concentrations of inducer IPTG (0.5 mM to 2.0 mM). This exercise was repeated several times, but with very little effect on intensity of band. Moreover, basal expression in uninduced sample i.e. without IPTG and decrease in expression with time, were some other problems in addition to inconsistency in results.

**Figure 2:**
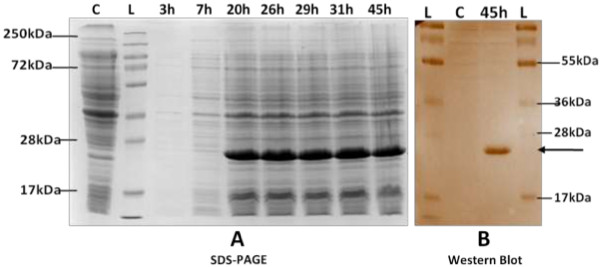
**Expression of recombinant protein in ZYP-5052 medium. A)**. Coomassie stained 12% SDS-PAGE of lysates shows proteins expressed in ZYP-5052 at 25°C at different incubation time. Equal culture densities (corresponding to 1 OD_600_ cells) were analysed in each lane. Cells grown to saturation in PG, a non-inducing growth medium were loaded as control. Lane L: Protein marker; Lane C: Control; Lane 3 h-45 h: Induced samples at different incubation time, as indicated on top of each lane. **B)**. Western blot of expressed protein. Expressed protein was transferred to nitrocellulose membrane at 30 V overnight at 4°C. After blocking in 3% BSA, membrane was treated with mouse raised anti-His antibody (1 : 500) and HRP-conjugated anti-mouse IgG antibody and developed with DAB. Bold arrow denotes distinct band in induced sample corresponding to expressed protein of 25 kDa size. Control used is culture grown to saturation in PG medium. No expression is seen in control. Lane L: Protein marker; Lane C: Control; Lane 45 h: Sample processed 45 hr post incubation at 25°C.

In order to overcome these limitations, we selected the auto-induction medium ZYP-5052 [17], supposed to be a better medium for growth. While using this medium, there was no use of IPTG and the cells were grown upto saturation point. After 45 hours of incubation, the products were analysed on SDS-PAGE. We found not only a clear band of 25 kDa, but a good yield of product and no further increase in yield with incubation time. The control sample did not show any band (Figure 2A). The results were also confirmed by western blotting (Figure 2B). The protein obtained using this protocol was used in all subsequent studies for solubility studies and western blot assays.

**Figure 3:**
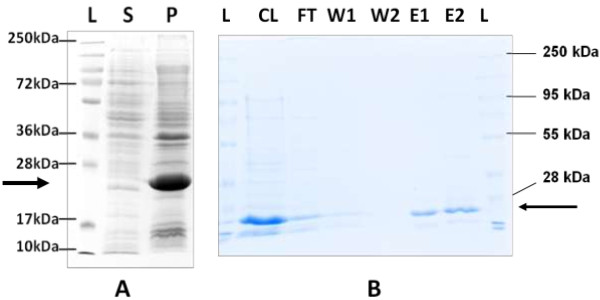
**Solubility and purification of expressed protein. A)**. Coomassie stained 12% SDS-PAGE of soluble and pellet fractions. Bold arrow denotes 25 kDa expression product. Lane L: Protein marker; Lane S: Protein from soluble fraction; Lane P: Protein from pellet/insoluble fraction. **B)**. Coomassie stained 12% SDS-PAGE of fractions obtained during Ni-NTA column purification. Bold arrow denotes 25 kDa expression product. Single bands were obtained in elution fractions. Lane L: Protein marker; Lane CL: Cleared lysate; Lane FT: Flow-through fraction; Lane W1: Wash I fraction; Lane W2: Wash II fraction; Lane E1: Elution I fraction; Lane E2: Elution II fraction.

The solubility of protein was tested by running of cleared lysate and pellet fractions on SDS-PAGE. Presence of protein in pellet fraction indicated its presence in insoluble part of cellular extract (Figure 3A). Solubilising agent (8 M Urea) was used to solubilize the target protein. The expressed protein was purified on Ni-NTA agarose column under denaturing conditions. The purity of protein was tested in eluted fraction using Coomassie stained SDS-PAGE. The band on SDS-PAGE corresponding to 25 kDa protein confirms the purified protein in eluate (Figure 3B).

**Figure 4:**
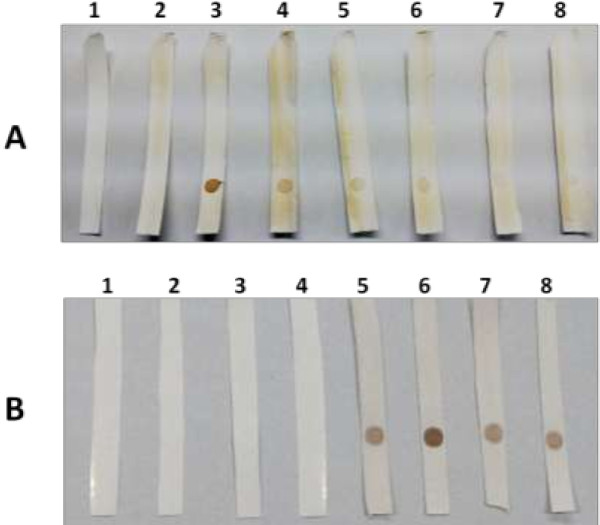
**Dot blot assay using human sera. A)**. Dilutions of sera in PBS were used as primary antibody. Positive control indicates the use of anti-His antibody to bind with antigen pre-coated on NC membrane. Serum without TTV-DNA and anti-His was used as negative control. Strip 1: PBS only; Strip 2: Negative control; Strip 3: Positive control; Strip 4: 1:500 sera dilution; Strip 5: 1:1000 sera dilution; Strip 6: 1:2000 sera dilution; Strip 7: 1:3000 sera dilution; Strip 8: 1:5000 sera dilution. **B)**. Dot blot assay using sera positive and negative for anti-TTV antibodies. Strips 1–4: Anti-TTV negative sera; Strips 5–8: Anti-TTV positive sera.

Subsequently, this purified protein was used to coat strips of nitrocellulose membrane to develop a dot blot assay and used for detection of anti-TTV antibodies in sera at varying dilutions. As shown in Figure 4A, no spot was observed in strip coated with PBS without antigen. Negative control sera also could not show any spot. Serum dilution of 1 : 1000 was found to be the last dilution for detection of anti-TTV antibodies (Figure 4A). Figure 4B demonstrates a total absence of spot in negative sera, thus indicating a high specificity of the developed assay. Based on this assay, a panel of sera from 88 patients with different renal and liver diseases and 25 healthy subjects were analysed for anti-TTV antibodies. The results in comparison to TTV-DNA are shown in Table 1. We observed a good coherence between TTV-DNA and anti-TTV antibodies in this panel of sera.

**Table 1:** Comparison of TTV-DNA with blot assay

**Disease group**	**No. Tested**	**TTV-DNA positivity**^**#**^		**N22 blot immunoassay**		**Anti-N22 positivity in DNA positive cases**	
		**No.**	**Percent**	**No.**	**Percent**	**No.**	**Percent**
**Chronic renal failure**	40	32	80.0%	33	82.5%	32/32	100%
Liver diseases*	48	27	56.3%	30	62.5%	27/27	100%
**Healthy controls**	25	16	64%	17	68%	16/16	100%

^**#**^TTV-DNA detection using N22 PCR.

*Liver diseases includes patients infected with Acute Viral Hepatitis (AVH), Chronic Viral Hepatitis (CVH) and Fulminant Hepatic Failure (FHF).

## Discussion

Torque Teno virus (TTV) infection is a benign infection with high prevalence in large number of healthy population reported worldwide [2,13]. Based on reports from several studies conducted world over, it appeared as TTV was simply a by-stander virus without causing a significant damage of tissue in human body [18,19]. Most of the patients carrying TTV infection alone do not present symptoms. After its characterization in 1997 [3], it was assumed that TTV may represent as one possible component of viruses causing non-A-G hepatitis. This assumption was also reinforced by the findings that TTV is predominantly present in liver tissue in higher levels as compared to its level in blood or other tissue [20]. However, later studies could not corroborate this virus to be responsible for causing liver diseases [19]. Our previous reports demonstrated its high prevalence both in healthy populations as well as patients with liver and renal diseases [16,21,22]. However, we could not observe TTV causing diseases symptoms or abnormal organ functions in these patients group. Few recent studies, of course, point towards its possible role in causing certain types of carcinoma [23-28]. TTV is a DNA virus and so, there is much of scope of its genome either to directly integrate with human genome or promote some mutation in cellular genome leading to oncogenesis. With all such possibilities, TTV is still a hot subject of study for various aspects including its possible involvement in oncogenesis. This definitely needs extensive studies on this micro-organism before it is cornered with ignorance assuming that it is a safer organism doing no harm to human body.

TTV infection is diagnosed by detection of TTV-DNA using polymerase chain reaction in sera of infected patients. However, due to high genetic variation in TTV sequences [12], there is still a need to develop a simple EIA system for detection of anti-TTV antibodies for diagnosis of TTV infection in all laboratories. Present study was planned with the impression that N22 region is a conserved domain in the coding region of the virus and so, its expression product may be a suitable agent for use as antigen in immunoassay.

N22 region of TTV genotype-1 was amplified using manually designed genotype specific primers and cloned in pGEM^®^-T Easy vector. Results of cloning were confirmed by sequencing and colony PCR. The sequence obtained was used for BLAST analysis and phylogenetic analysis revealing 97% identity of cloned product with known TTV genotype-1 isolate from databases. This confirms that amplified sequence corresponds to N22 region of TTV genotype-1. Re-amplification was performed using primers containing restriction sites to assist cloning in pET-28a(+) expression vector. The translation product was expressed as a His-tagged fusion protein in recombinant *E. coli* cells. The size of expression product was determined using SDS-PAGE analysis and found to be 25 kDa. This was further verified by western blotting on nitrocellulose membrane. This is the first report demonstrating expression of full length N22 region for its use in developing immunoassay. All earlier studies demonstrated the expression of ORF2 and used the product in developing immunoassay [12,29]. Possibly, attempts were made in ORF1 [5,15,30] but could not be successful. We envisaged more prospects in use of N22 region for developing a better immunoassay system. The recombinant protein was purified using metal-chelate affinity chromatography, as used in other studies [12]. This purified expressed protein was used to develop a blot based immunoassay to analyse the presence of TTV antibodies in human sera. Analysis of 75 sera positive for TTV-DNA showed a clear spot on nitrocellulose membrane when analysed by this blot assay. On the contrary, sera negative for TTV-DNA did not show presence of anti-TTV antibodies. This shows high specificity of the present assay system. We achieved better rates of detection using N22 based immunoassay as compared to PCR method. This suggests that the immunoassay also picked response in sera with possible past infection, or with viral load too low to be detectable by PCR method, as suggested in other studies [5]. This again indicates that use of translational product from N22 region is a better method to detect TTV infection.

## Conclusions

This demonstrates that the blot assay developed using N22 region could be used for an effective screening of anti-TTV antibodies in sera from patients as well as healthy individuals. The system developed has an advantage over PCR based methods as it does not involve use of expensive equipments and can be easily performed in small laboratories. Also, this method is useful as it determines past infection of TTV which is not possible using DNA amplification based methods. Hence, it may be concluded that the immunoassay developed serves its purpose as an easy and simple method to detect TTV infection in human sera.

## Materials and methods

### Serum samples

Sera were collected with informed consent from 88 adult patients with liver and renal diseases attending the Outpatient Department at All India Institute of Medical Sciences, New Delhi. Liver disease group included patients with acute viral hepatitis (AVH), chronic viral hepatitis (CVH) and fulminant hepatic failure (FHF), while renal disease group included patients with chronic renal failure (CRF). These patients were diagnosed on the basis of accepted clinical, and biochemical criteria. Twenty five age and sex matched healthy subjects were used as controls. 6–10 mL of venous blood was drawn and aliqouted in plain tubes without any anticoagulant. Sera were separated from whole blood after centrifugation and stored at -70°C until further analysis. Repeated freezing and thawing of serum was avoided as far as possible.

### Detection of DNA and genotyping of TTV

Sera were analysed for the presence of TTV-DNA and subsequent genotyping of all TTV-positive sera were carried out using RFLP as detailed in our previous publication [16]. Total DNA was purified from 200 μl of sera using High Pure Viral Nucleic Acid kit (Roche Applied Science, Germany) following manufacturer’s instruction. The extracted DNA was used as template for PCR amplification. All steps were performed under laminar air flow hoods using disposable racks and aerosol-resistant tips to avoid sample to sample cross-contamination.

### Amplification of full length N22 region

Full length N22 region of TTV genotype-1 was amplified from sera using Taq PCR Core kit (Qiagen, Germany). Primers used were: 5′- ATGTCCTACTTTGAAA -3′ (forward) and 5′- TTACCAGATCCACTTA -3′ (reverse), and were designed using JustBio (http://www.justbio.com) and Primer3 software (http://primer3.ut.ee/). The PCR reactions (25 μL) contained 1X Q-buffer, 1X PCR buffer, 10 mM of each dNTP, 25 pMole of each primer, 2 mM MgCl_2_ and 0.75U of Taq Polymerase. The PCR cycles consisted of : 94°C for 5 min; 35 cycles of 94°C for 30 sec, 42°C for 45 sec, 72°C for 45 sec; followed by final extension at 72°C for 5 min. Second round comprising 25 cycles was performed under the same conditions using 5 μL of the first round product. The amplified DNA was electrophoresed on 1.2% agarose gel stained with ethidium bromide (EtBr) and identified as ~500 bp amplicon after exposure to UV light.

### TA cloning and sequence analysis

The ~500 bp amplicon corresponding to N22 region was excised and extracted with the QIAquick Gel Extraction kit (Qiagen, Germany). The purified PCR product was cloned using commercial vector kit (Promega Corporation, WI, USA). Reaction mixture contained 50 ng pGEM®-T Easy vector, purified amplicon, 1X ligation buffer and 3 Weiss units of T4 DNA Ligase and resulting plasmids were transformed into *Escherichia coli* DH5α cells (Invitrogen, USA) by heat shock method. The transformed cells were grown on LB-agar plates containing 100 μg/mL ampicillin (Sigma-Aldrich, USA). Insertion was confirmed by colony PCR and sequencing using T7 and target specific primers, as described in our previous studies [29,31]. Database searches were performed using NCBI BLAST (http://www.ncbi.nlm.nih.gov/BLAST). Phylogenetic analysis was performed using neighbour joining algorithm of PHYLIP program package (http://evolution.gs.washington.edu/phylip.html). Data set was bootstrap re-sampled 1000 times to ascertain support for major branches of the tree.

### Cloning in expression vector

To facilitate cloning into expression vector, the N22 region cloned in pGEM^®^-T vector was re-amplified using sense 5′-TACTAC*GGATCC*ATGTCCTACTTTGAA-3′ and anti-sense primer 5′- AACTAT*GAGCTC*TTACCAGATCCACTTA-3′ containing *BamH*I and *Sac*I restriction sites (shown in italics) at the 5′ and 3′ ends respectively. The amplification conditions were same as used earlier, except for annealing step that was performed at 55°C for 45 sec. The PCR product was digested sequentially using restriction enzymes *BamH*I and *Sac*I (NEB, USA) and ligated into pET-28a(+) expression vector (Novagen, Germany). The recombinant vector was transformed in *E. coli* DH5α cells and screened using LB-agar media plates containing 50 μg/mL kanamycin (Sigma-Aldrich, USA). Positive clones were confirmed by colony PCR and bi-directional sequencing. Plasmid from selected recombinant clones was extracted using Qiagen Plasmid Mini kit (Qiagen, Germany).

### Expression of TTV protein

N22 region was subsequently expressed as a fusion protein containing hexa-histidine tag (His6-G1N22-pET-28a) in *Escherichia coli* BL21 cells (Invitrogen, USA) using LB growth medium. Isopropyl-ß-D-thiogalactoside (IPTG) at 0.5– 2.0 mM concentrations was used for 2, 4, 6, 8 and 20 hour inductions, both at 25°C and 37°C. Parallel cultures, without addition of IPTG, were used as controls. Cells from induced and control setups were harvested by centrifugation at 10,000 g for 5 min at 4°C. Whole-cell lysates were studied by 12% SDS-PAGE [32] and protein bands were visualized with Coomassie stain (SRL, India).

### Expression in ZYP-5052 auto-induction medium

Attempts were made to conduct expression in auto-induction medium also. Principle of this medium is based on presence of carbon sources in the medium that are metabolized differentially to promote high density cell growth and automatically induce protein expression from lac promoters. The medium contains both glucose and lactose as the carbon source. Lactose, often present in undefined components of complex media such as Tryptone, has been shown to cause unintended, sporadic induction of expression in LB media. This can lead to basal expression of target protein, even without addition of IPTG [17].

Use of auto-induction medium was envisaged for a better growth/yield of expression product. For this, freshly screened colonies were grown to saturation in a non-inducing defined medium (PG) at 37°C and this saturated culture was used to inoculate ZYP-5052, a complex auto-inducing growth media containing 100 mM phosphates and 150 μg/ml kanamycin and incubated at 25°C with shaking at 200 rpm. Aliquots were drawn at regular intervals and culture was grown to saturation. To check the time course production of recombinant protein, cells corresponding to 1 OD cell density were harvested, re-suspended in 4X SDS-PAGE sample buffer and analysed on SDS-PAGE. Cells grown to saturation in PG growth medium were used as control. Final culture was harvested by centrifugation at 10,000 g for 5 min at 4°C. The pellet was retained and stored at -80°C till further analysis.

### Immunoblotting

Proteins from SDS-PAGE were transferred electrophoretically to nitrocellulose membrane (Genetix, India) using mini-transblot (Biorad, USA) at 30 V overnight at 4°C [33] and the membrane was blocked with 3% BSA (Amresco, USA) in PBS (137 mM NaCl, 2.7 mM KCl, 10 mM Na_2_HPO_4_, 2 mM KH_2_PO_4,_ pH 7.4). For immunodetection of the His fusion proteins, the primary antibody used was His probe (H-3) mouse monoclonal IgG (1 : 500; Santa Cruz Biotechnology, USA) and the secondary antibody was HRP-conjugated anti-mouse IgG antibody (1 : 2000; Santa Cruz Biotechnology, USA). Bound antibodies were detected using di-aminobenzidine (DAB) (Amresco, USA) as substrate.

### Purification of expressed N22 protein

After confirmation on western blot, protein was expressed on large scale. 500 mL culture of recombinant *E. coli* BL21 was grown in ZYP-5052 medium till saturation. The culture was harvested 45 hours post-induction by centrifugation at 10,000 g for 5 min at 4°C and pellet was re-suspended in ice-cold lysis buffer (50 mM NaH_2_PO_4_, 300 mM NaCl, 10 mM Imidazole, pH 8.0). Sonication was used to lyse the cells (six 10 sec bursts/ 10 sec cooling cycles at 200 W in a Branson Sonicator, USA). Cleared lysate obtained after centrifugation (10,000 g for 20 min at 4°C) was separated from cellular debris (pellet). Both, cleared lysate (soluble protein fraction) and pellet (insoluble protein fraction), were analysed on SDS-PAGE to determine whether recombinant protein is expressed in soluble form or as aggregate in inclusion bodies. To solubilize recombinant protein present in inclusion bodies, 8 M Urea in 100 mM NaH_2_PO_4_, 10 mM Tris-Cl, pH 8.0 was used. Purification was performed using Ni-NTA agarose columns (Qiagen, Germany) under denaturing conditions using manufacturer’s instructions. The lysate obtained after treatment with 8 M urea was incubated with Ni-NTA agarose for 30 min on a rotary shaker at room temperature and loaded into an empty column. Flow-through obtained was collected and the column was washed twice with 2 bed volumes of wash buffer (100 mM NaH_2_PO_4_, 10 mM Tris-Cl, 8 M Urea, pH 6.3) to remove non-specific proteins. The bound His-tagged recombinant protein was eluted in two steps from column using elution buffer (100 mM NaH_2_PO_4_, 10 mM Tris-Cl, 8 M Urea, pH 4.5). Protein concentration in all fractions obtained were determined using Bradford’s reagent [34] and subsequently analysed on 12% SDS PAGE.

### Development of blot assay

The purified protein was dialyzed to remove urea and subsequently coated on nitrocellulose membrane (50 mm × 5 mm). The control strip was coated with PBS only. After drying, the strips were blocked with 3% BSA in PBS followed by incubation overnight at 4°C. Human sera serially diluted in PBS (1: 500, 1: 1000, 1: 2000, 1: 3000 and 1: 5000) were used as primary antibody for detection of anti-TTV antibodies. A serum without TTV-DNA (1: 500 dilution) was used in negative control. Diluted sera were added to the NC strips and incubated at room temperature for 2 hours with constant shaking. Secondary antibody (goat anti-human IgG-HRP conjugated antibody; Santa Cruz Biotechnology, USA) was added in 1: 4000 dilution in PBS and incubated for 2 hours at room temperature. The strip treated with anti-His (His probe H-3) primary antibody was used as positive control. The blot was developed using HRP-conjugated anti-mouse IgG antibody as detailed earlier. Finally, the substrate (DAB) was used for detection of antigen-antibody complex in all strips. The development of colour in the coated region of strips confirms the binding of anti-TTV antibodies to expressed N22 translational product.

### Detection of anti-TTV antibodies in human sera

Panel of sera from liver and renal disease patients as well as healthy individuals were analysed using this blot assay. All sera from patients were used at a dilution of 1: 1000 simultaneously using control sera at a dilution 1: 500. The presence of dot blot on nitrocellulose membrane was used as an indication of anti-TTV antibodies in serum. The results of anti-TTV immunoassay were compared with presence of TTV-DNA in same panel of sera.

### Ethics statement

The study was approved by the Ethics Committee of All India Institute of Medical Sciences, New Delhi.

## Abbreviations

TTV: Torque teno virus; PCR: Polymerase chain reaction; ORF: Open reading frame; RFLP: Restriction fragment length polymorphism; UV: Ultra-violet; LB: Lysogeny broth; OD: Optical density; DAB: di-aminobenzidine; PBS: Phosphate buffered saline; NC: Nitrocellulose; CRF: Chronic renal failure.

## Competing interests

The authors declare that they have no competing interests.

## Authors’ contributions

DSM performed the experiments, analysed the results and prepared the manuscript. MI conceived the study, participated in its design and edited the manuscript. Both authors read and approved the final manuscript.

## Supplementary Material

Additional file 1: Figure S1 Colony PCR using genotype specific primers containing restriction sites. Successful transformation and cloning into pET-28a(+) vector demonstrated by presence of ~500 bp band in 1.2% agarose gel corresponding to N22. Lane 1: No template control; Lane 2: 50 bp DNA marker; Lane 3–6: Amplification of ~500 bp corresponding to N22 region amplified from colonies of *E. coli* following transformation. Click here for file
