# Supplementary figures and images for: Cloning and expression of N22 region of Torque Teno virus (TTV) genome and use of peptide in developing immunoassay for TTV antibodies
Source: Virol J. 2014 May 20;11:96. doi: 10.1186/1743-422X-11-96 (PMC4032458; doi:10.1186/1743-422X-11-96)

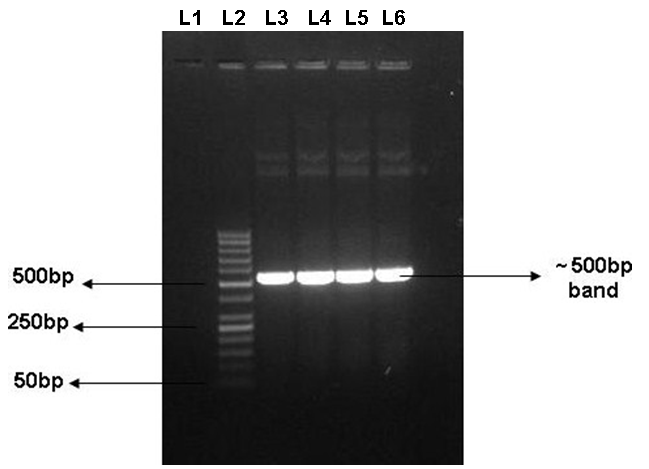

Supplement: Additional file 1: Figure S1 — Colony PCR using genotype specific primers containing restriction sites. Successful transformation and cloning into pET-28a(+) vector demonstrated by presence of ~500 bp band in 1.2% agarose gel corresponding to N22. Lane 1: No template control; Lane 2: 50 bp DNA marker; Lane 3–6: Amplification of ~500 bp corresponding to N22 region amplified from colonies of E. coli following transformation. [file 1743-422X-11-96-S1.docx]
